# Morbidity due trauma in children of the community of Paraisopolis, São Paulo, Brazil

**DOI:** 10.1590/S1679-45082014AO2434

**Published:** 2014

**Authors:** Renata Dejtiar Waksman, Renato Melli Carrera, Erica Santos, Sulim Abramovici, Cláudio Schvartsman

**Affiliations:** 1 Hospital Israelita Albert Einstein, São Paulo, SP, Brazil

**Keywords:** Accidents, Wounds and injuries, Socioeconomic factors, Child

## Abstract

**Objective:**

To identify the factors that determine unintentional injuries in children living in the community of Paraisopolis, in the city of São Paulo, Brazil.

**Methods:**

A cross-sectional and non-controlled study. Data collected during 4 consecutive months through questionnaires filled out for the Einstein Program in Paraisopolis Community included identification of the patient and his/her family, scholarity level, housing conditions, storage of hazardous products, access to the streets and concrete slab ceilings, supervision, and trauma mechanism involved. The observed data were treated as absolute and relative frequencies; χ^2^, Fisher's exact test, Student's *t* test, and Mann-Whitney's tests were implemented, with a significance level of 5% (p<0.05).

**Results:**

A total of 1,490 questionnaires were analyzed. There was a predominance of trauma within boys (59.6%) and the medium age was 5.2 years. The predominant educational level of the parents was incomplete junior school education. The main caregiver identified was the mother (69.4%). Among the children that suffered trauma, 56.4% belonged to large families (≥6 people), lived in houses up to three rooms, and a family income up to R$ 1,000.00 (76.6%). Easy access to hazardous materials was considerable and free access to concrete slab ceilings was reported in 92.8% of the cases. The main trauma mechanisms were falls and burns. In this study, the child victim of a fall was aged under 5 years.

**Conclusion:**

Small children that live in a hazardous environment have a significant tendency to suffering trauma

## RESUMO

**Objetivo:**

Identificar os fatores determinantes de lesões não intencionais na população pediátrica residente na comunidade de Paraisópolis, em São Paulo.

**Métodos:**

Estudo transversal, não controlado. Os dados coletados durante 4 meses consecutivos, por meio de questionários preenchidos para o Programa Einstein na Comunidade de Paraisópolis foram: identificação do paciente e familiares, escolaridade, condições da moradia, armazenamento de produtos perigosos, acesso à rua e à laje, supervisão e mecanismo de trauma envolvido. Os dados observados foram tratados por meio de frequências absolutas e relativas; foram empregados os testes χ^2^, exato de Fisher, *t* de Student Mann-Whitney, com nível de significância de 5% (p<0,05).

**Resultados:**

Foram analisados 1.490 questionários. Houve predominância de traumatismos entre meninos (59,6%) e a média de idade foi de 5,2 anos. Ensino Fundamental incompleto foi o nível de escolaridade predominante entre os pais. O cuidador principal identificado foi a mãe (69,4%). Das crianças que sofreram trauma, 56,4% pertenciam a famílias numerosas (≥6 pessoas), moravam em casas de até três cômodos com renda familiar de até R$ 1.000,00 (76,6%). O acesso fácil a substâncias perigosas foi considerável, e o livre acesso a lajes foi relatado em 92,8% dos casos. Os principais mecanismos de trauma foram quedas e queimaduras. Neste estudo, a criança vítima de queda tinha menos de 5 anos de idade.

**Conclusão:**

Crianças pequenas que vivem em um ambiente de risco apresentam tendência significativa a sofrer eventos traumáticos.

## INTRODUCTION

Trauma has gained expression in various centers of the world as a result of mortality and the temporary or permanent impairment it generates, regardless of age group, gender, ethnicity, creed, or social stratum.

The main causes of death in children less than 18 years of age are road accidents, drowning, burns, falls, and poisoning. It is also known that 95% of the events occur in countries with low and medium incomes.^(1)^

In Brazil, in 2009, according to the Data Bank of the public Unified Healthcare System (DATASUS),^(2)^ 25% of admissions by SUS were related to those younger than 20 years of age, and 95% of them were due to trauma.

Various socioeconomic factors contribute towards a greater occurrence of these events involving children in poor communities, since they do not have access to areas and resources where they can safely play, and because they generally live under dangerous conditions (houses at greater risk of fires, unprotected windows, unsafe guardrails and stairs, or near areas with intense road traffic).^(1,3)^

The mechanisms and lesions that generate disability in our environment are difficult to define, since each region has its peculiarities and there is a considerable correlation between the distribution of cases and the daily routine of the communities less favored, it is important to evaluate the behavior of the different non-intentional external causes in these communities.^(1,3,4)^

## OBJECTIVE

To identify the different socioeconomic factors determining the most prevalent non-intentional injuries in a population of children living in the Paraisopolis community, in the city of São Paulo.

## METHODS

As cross-sectional non-controlled study in which data were collected during four consecutive months (September to December 2007). The database for analysis was composed of the questionnaires completed at the outpatient clinic, located in the poor community that takes part in the Einstein Program in Paraisopolis Community (PECP, abbreviation in Portuguese).

The community of Paraisopolis has a high population density, with an estimated population of 80 thousand inhabitants, divided amongst 17730 houses, in which 78% of the people have a steady job in the region. The mean family monthly income at the time of the study was R$ 614.00.^(5)^

Before initiating the data collection stage by means of interviews with the families or guardians, four undergraduate students in the second year of the Nursing School of the *Sociedade Beneficente Israelita Brasileira Albert Einstein* (SBIBAE) were involved and trained to gather data. The students were trained to select the families that were in the waiting area for routine visits at the PECP outpatient clinic, from Monday to Friday, in the morning and afternoon periods, during four months of the study.

The people interviewed at the time of selection, whose children had suffered any kind of trauma during the 20 month period that preceded the research, were considered eligible and agreed to participate in the individualized interview.

The inclusion criteria considered were presence of a traumatic event, assistance and outpatient follow-up at the PECP (which guarantees the place of residence in that locality), and age <15 years.

Data were collected on identification of the patient, the family (parents, siblings, other residents in the same house), schooling level, living conditions, presence and location of hazardous products, access to the concrete ceiling slab, access to the surrounding streets, supervision, relevant past medical history, and mechanism of the trauma involved.

The data were tabulated for analyses. Minitab Statistical 16^®^ and Statistical Package for Social Sciences software were used. Data were presented as absolute and relative frequencies.

To verify the association between the two qualitative variables, χ^2^ test or Fisher's exact test were applied – in the case of 2 × 2 tables. For the association between a dichotomous and a numeric variable, with normal distribution, the Student's *t* test or Mann-Whitney's test were used – in the case of non-normal distribution. The level of significance adopted was 5% (p<0.05).

The questionnaire used was previously submitted for approval to the Clinical Research Committee of the *Instituto Israelita de Ensino e Pesquisa* (CEP no. 07/711). Each interview was only conducted after detailed explanations as to the objectives of the study and signing of the Informed Consent Form by the person responsible for the information given.

## RESULTS

A total of 1,490 questionnaires were analyzed relative to the children and adolescents aged under 15 years, who presented with at least one traumatic event over the previous 20 months (January 1st, 2006 to August 31st, 2007).

There was a greater incidence of trauma among boys (59.6%; p<0.001). The mean age was 5.2 years (3 months to 14.8 years), and the median age was 4.9 years.

The mean age of the parents was 32.4 years, of the mothers 29.5 years, and of the siblings, 8.3 years. The predominant schooling level of the parents was incomplete junior school (54.5% for fathers and 52.4% for mothers).

The mean number of siblings was 1.4 (zero to six siblings in the 1490 interviews; their mean age was 8.3 years (zero to 33 years).

As to the caregiver, this role was filled either by the mother (69.4%), or by the grandmother or aunt (11.9%) (Table 1).

**Table 1:** Demographic profile of the children and adolescents among the study participants

Demographic profile			n	%	Mean (min-max)
Target child			1,490	100.0	
Gender					
	Male		888	59.6	
	Female		602	40.4	
Age					
	Target child		1,490		5.2 (3 m-14.8 y)
	Father		1,490		32.4 (17-63 y)
	Mother		1,490		29.5 (13-54 y)
	Siblings		1,490		8.3 (0-33 y)
Number of siblings					1.4 (0-6)
Predominant schooling level					
	Father				
		Incomplete junior school	740	54.5	
		Complete high school	201	11.5	
	Mother				
		Incomplete junior school	764	52.4	
		Complete high school	213	16.9	
Caregiver					
	Mother		1012	69.4	
	Grandmother/aunt		174	11.9	

m: months; y: years; min: minimum; max: maximum.

As to the living accommodations of those interviewed, in general, 4.6 persons lived in the same house, with three rooms, on average, and 1.1 bathrooms per house. Of the children that belong to large families (more than 6 people), 56.4% lived in small houses – with up to three rooms (Table 2).

**Table 2:** Number of persons, rooms, and bathrooms per household among the study participants

	n	Mean (min-max)
Number of persons	1,476	4.62 (3-15)
Number of rooms	1,474	3.04 (1-10)
Number of bathrooms	1,471	1.08 (0-5)

min: minimum; max: maximum.

The family income was the result of paid work basically earned by the father (77%) and by the mother (52.2%), with a family monthly income range of up to R$ 1,000.00 in 76.6% of the cases.

**Figure 1:**
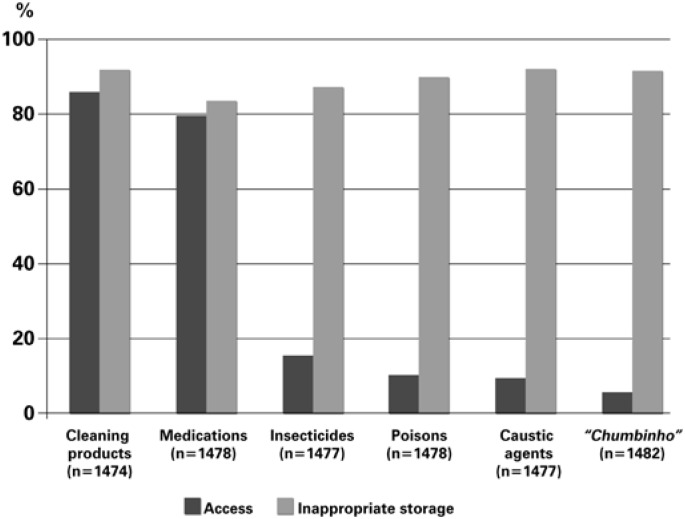
Access of children to and storage of hazardous substances, as per interviews with the caregivers

Easy access to various substances, such as medications (79.8%) and cleaning materials (86.1%), rat and cockroach poison (such as one named “*chumbinho*”), and known poisonous plants. Storing of products was considered inappropriate or undefined in more than 80% of the cases. For caustic agents, insecticides, and poisons to control animal diseases, although the access was relatively low, inappropriate or undefined storage was expressive (Figure 1).

As to access to the concrete ceiling slabs, of the 926 interviewees that answered this question, 92.8% informed that the access to this structure is free and only 31.1% confirmed the use of protection or isolation of that area.

Among the primary mechanisms of trauma, falls (77.98% of the events) and burns (15.36%) stood out, followed by mechanisms less represented in this study, such as being hit by vehicles, bites, collisions, intoxications, and drowning (Table 3).

**Table 3:** Primary mechanisms of trauma

Trauma mechanism	n (%)
Falls	1,162 (77.98)
Burns	229 (15.36)
Being hit by a vehicles	76 (5.10)
Collisions	67 (4.50)
Animal bites	67 (4.50)
Bicycle	65 (4.36)
Intoxications	46 (3.10)
Drowning	7 (0.50)
Other events	90 (6.04)

Among the children that suffered falls, the mean age was lower when compared to those who suffered any other types of trauma (4.92 for falls *versus* 6.06 years for other traumas; p<0.001).

## DISCUSSION

Non-intentional lesions represent the predominant group of causes of death in children as of 1 year of age, and the third cause of hospitalization of children and adolescents in Brazil.^(2,3,6)^

Some beliefs of the adults regarding the capacity of children to deal with danger, inevitability of destiny, and the influence of cultural standards and behavior place the children at risk and interfere in a technically acceptable approach.^(3,7–11)^

Traumatic lesions occur in a great proportion in boys, according to what was identified in this study, and boys can have twice as many chances of suffering injuries as girls after the first year of life – a difference that increases with age.^(3,12)^ In developed countries, the general average is 1.3 times more frequent among representatives of the male gender under the age of 20 years.^(13)^ In this study, similar to what has been described in the literature, boys were more likely to have had traumatic events in their past history.

Other characteristics of a child, such as age and phase of development, may also influence the occurrence of lesions, besides contributing to increases exposure.^(8,9)^ Nothing in peculiar was identified as to age in this current study.

The supervision of small children is widely known as a vital protection factor against risks of the home environment. There is considerable indirect evidence associating supervision and the risk of trauma.^(14)^ Children left at home alone or in the company of other children, without appropriate care and supervision, run a higher risk than other children, cared for by an attentive adult.^(11)^ It was not possible to associate here the absence of supervision with the greater propensity towards trauma, since it was not relevant. Supervision (or lack of it) and the moment of the traumatic event were not specifically investigated in this study.

However, we point out that the children identified by means of an investigation live in environments full of risks, with easy access to the stove, ceiling slab, and streets. As to chemical substances, easy access was identified in the majority of cases, as well as inappropriate storage.

Several different factors contribute towards the increased risk of lesions in children, such as large families, number of persons living in the same household, poverty, and unemployment.^(7,15–17)^ The socioeconomic disadvantage (represented by poverty, low level of scholarity, and material privation) demonstrably are related to the increased morbidity and mortality determined by external non-intentional causes.^(7,18–21)^

This study identified some of these factors, such as large families, small number of rooms in the house, and socioeconomic disadvantages.

Added to that are differences in perception and sense of responsibility of the parents as to the children's safety. Lack of safety, non-installation of equipment for protection (in windows, stairs, and ceiling slabs), inadequate maintenance, and lack of areas destined for play are considered important by the families interviewed, reflecting different types of exposure of the children to various risks. ^(3,4,8,22,23)^

The epidemiological distribution of trauma mechanisms that lead to mortality differs from what is usually found for lesions related to morbidity. In decreasing order of importance, there are falls, burns, and other trauma mechanisms.

For children aged between zero and four years, falls represent one the greatest causes of lesions and trauma in the world.^(4)^ Although about 10% of the children live in high-income countries, most epidemiological studies published on the epidemiology of wounds related to falls in children were carried out in these countries. Worldwide statistics on non-fatal falls were identified as a gap to be filled in this area.^(4,24,25)^

There is no single prevention measure that has obtained success alone. According to the report by the World Health Organization/United Nations Children's Fund (OMS/UNICEF),^(1)^ six basic principles, when applied together, increase the chance of success in preventing non-intentional lesions and trauma in children: legislation, with regulation and reinforcement; modyfing products; environmental modification; home visits; promotion of safety devices; and education in and training of skills.

Just as important as knowing what works is the notion of what should be avoided. Preventive strategies applied in developed countries may or may not generate the same results or beneficial effects if tested in other countries; some may even determine negative consequences. The Brazilian society is still in the situation of epidemiological transition, in which proportional mortality by injuries is still growing, and its control starts to be successful.^(3)^

There is need for serious research in our country, which should help determine the best combination of strategies to prevent and treat multiple risks of trauma along childhood, so that measures can be implemented to diminish these indices in a concrete manner, besides guaranteeing that the lessons learned might help others that are similar.

And what should be the best interference to modify this process? The combination of basic recommendations based on evidence with the practical expertise and specific experience within the current regional and national context.

## CONCLUSION

Since there is a strong association between increased morbidity and socioeconomic disadvantages, preventive interventions, based on solid evidence, associated with regional reality should address the exposure to dangers that affect these children in a disproportional way.
